# Regulation of the epithelial sodium channel [ENaC] in kidneys of salt-sensitive Dahl rats: Insights on alternative splicing

**DOI:** 10.1186/1755-7682-2-28

**Published:** 2009-09-29

**Authors:** Marlene F Shehata

**Affiliations:** 1 Department of Cellular and Molecular Medicine, Faculty of Medicine, University of Ottawa, Ottawa, ON, Canada

## Abstract

The epithelial sodium channel [ENaC] is critical for the maintenance of sodium balance, extracellular fluid volume and long term blood pressure control. Monogenic disorders causing ENaC hyperactivity have led to a severe form of hereditary hypertension in humans, known as Liddle's syndrome. Similarly, in animal models, ENaC hyperactivity has been well documented in kidneys of salt-sensitive [S] Dahl rats [a genetic model of salt-sensitive hypertension] *versus *their normotensive control [Dahl salt-resistant [R] rats]. The purpose of the present review is to highlight the differential regulation of ENaC in kidneys of Dahl S *versus *R rats. A systematic overview of the putative role of alternative splicing of the main α subunit of ENaC [α ENaC] in modulating ENaC expression in kidneys of Dahl rats will be discussed. Finally, a better understanding of the meaningful contribution of ENaC in the pathogenesis of salt-sensitive hypertension will be achieved upon completion of this review.

## Salt-sensitive hypertension

Over one-fifth of Canadian adults are diagnosed with hypertension  and over 50% of primary hypertensive patients are salt-sensitive [1]. Despite the fact that hypertension is the primary risk factor for stroke and heart disease, and has been labeled by the "silent killer disease", yet 42% of Canadians are still unaware of their increased blood pressures .

These above statistics, combined with the realization that salt-sensitive hypertension exacerbates mortality rates [2], worsens manifestations of target organ damage [3,4] and is a common finding in aging populations, emphasize the importance of identifying novel targets for prevention and treatment of salt-sensitive hypertension.

The major contributor to the pathogenesis of salt-sensitive hypertension is dietary salt [5]. Dietary sodium, in turn, has sodium chloride [NaCl] as its major constituent. The sodium ion [Na^+^] is transported into the superficial cells of several organs (see below) primarily via the amiloride-sensitive Epithelial Sodium Channel [ENaC]. Owing to the fact that inadequate Na^+ ^excretion is a risk factor for hypertension, ENaC represents an attractive therapeutic target to study in salt-sensitive hypertension and α ENaC regulation by alternative splicing will be the focus of the present review.

## ENaC α, β, and γ as candidate genes for blood pressure regulation

ENaC is highly selective for Na^+ ^and mediates Na^+^- entry [down an electrochemical gradient] through the apical membrane of renal epithelial cells. ENaC also regulates sodium transport in other epithelia such as the alveolar epithelium, distal colon, brain, salivary duct and sweat glands [6-8]. Additionally, ENaC has proved essential for lung fluid clearance in newborn mice [8] and the entire salt taste perception in rodents.

Although the ENaC accounts for a small proportion of renal sodium reabsorption [<5%], nevertheless it still constitutes the rate-limiting step of sodium reabsorption in the distal nephron. The control of Na^+ ^movements in these epithelia is critical for the regulation (or homeostasis) of extracellular fluid volume, electrolyte balance and long term blood pressure.

One of the major breakthroughs in understanding the central role played by ENaC in blood pressure regulation was the demonstration of linkage between the ENaC and a rare form of hereditary severe salt-sensitive hypertension [Liddle's syndrome] [9]. Gain-of-function mutations and/or truncations in ENaC α, β and γ genes have been identified in patients with Liddle's syndrome. Later on, transgenic mouse models engineered with Liddle's mutations confirmed the critical role of ENaC in blood pressure regulation [10].

In contrast, loss-of-function mutations in the α and β subunits of ENaC have been identified in patients with pseudohypoaldosteronism, a salt-wasting nephropathy that results in defective sodium transport in many organs containing the ENaC, such as the kidney, lung, colon, sweat and salivary glands.

In summary, ENaC serves as an attractive candidate gene to study in salt-sensitive hypertension for the following reasons: i] ENaC serves as a key channel in controlling the rate of renal sodium reabsorption [7], ii] Genetic defects causing ENaC hyperactivity have led to a monogenic form of hereditary hypertension in humans [Liddle's syndrome]. This suggests that salt-sensitivity might arise from subtle defects in ENaC function and/or regulation [11]. iii] Moreover, ENaC activity is twice as high in renal collecting ducts of high salt-fed genetically predetermined salt-sensitive Dahl S rats *versus *their normotensive controls [Dahl R rats] that remain resistant to salt-sensitive hypertension on high salt diet [12], iv] Finally, ENaC blockade in the brain by benzamil rescued Dahl S rats from salt-induced hypertension [13]. Therefore, owing to the established importance of ENaC in blood pressure regulation, and in an attempt to understand the genetic differences in ENaC among Dahl S and R rats, the present review will highlight the putative mechanisms of ENaC regulation via alternative splicing. A comprehensive review of ENaC structure, function and differences in Dahl S *versus *R rats will be presented in details as a prelude to alternative splicing regulation of ENaC.

## Structure of ENaC

**Figure 1:**
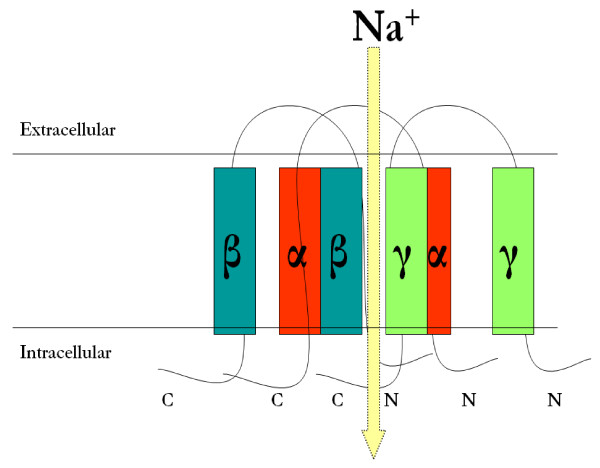
**Structure of the Epithelial Sodium Channel [ENaC]**. The amiloride-sensitive epithelial sodium channel [ENaC] is composed of three homologous α, β and γ protein subunits of corresponding 698, 638 and 650 amino acids in length [14,15]. ENaC α, β and γ subunits share approximately 30% homology at the amino acid level and each subunit correspond to a molecular mass of 70-80 kDa. The three ENaC subunits are inserted into the plasma membrane with a proposed stoichiometry of 2:1:1 [16] as shown in the above figure or 3:3:3 [18]. Each ENaC protein subunit is formed up of four major domains: the cytoplasmic N terminus, the large extracellular loop, the two short hydrophobic segments known as the transmembrane domains 1 and 2 [TM1 and 2] and the cytoplasmic C-terminus. The N- and C-termini face the cytosolic side, while the extracellular loop faces the extracellular side [19]. All three subunits cooperate to form the channel pore via the transmembrane domains.

The amiloride-sensitive epithelial sodium channel [ENaC] is composed of three homologous α, β and γ protein subunits of corresponding 698, 638 and 650 amino acids in length [14,15]. ENaC α, β and γ subunits share approximately 30% homology at the amino acid level and each subunit correspond to a molecular mass of 70-85 kDa. The three ENaC subunits are inserted into the plasma membrane with a proposed stoichiometry of 2:1:1 [16] or 3:3:3 [17,18]. The structure of the ENaC is found in figure 1.

Each ENaC protein subunit is formed up of four major domains: a cytoplasmic N-terminus, a large extracellular loop, two short hydrophobic segments known as the transmembrane domains 1 and 2 [TM1 and 2] and a cytoplasmic C-terminus. The N- and C- termini face the cytosolic side, while the extracellular loop faces the extracellular side [19]. The channel domains are important for basic channel function such as the translocation of Na^+^- ions across the membrane and for the modulation of ENaC activity at the cell surface. All three subunits cooperate to form the channel pore.

Among the channel domains the C-terminus has gained considerably high attention because almost all mutations discovered so far affecting the C-terminus cause a rare form of hereditary hypertension called the Liddle's syndrome. These mutations target the PY motif [PPPXY, where P = proline, X = any amino acid and Y = tyrosine] within the intracellular C-termini of the three subunits [20,21]. The PY motif provides a mechanism for enhancing ENaC retrieval from the plasma membrane. Therefore, mutations of the PY motif prolong the half-life of the channel at the cell surface as a result of impaired internalization of ENaC [21].

**Figure 2:**
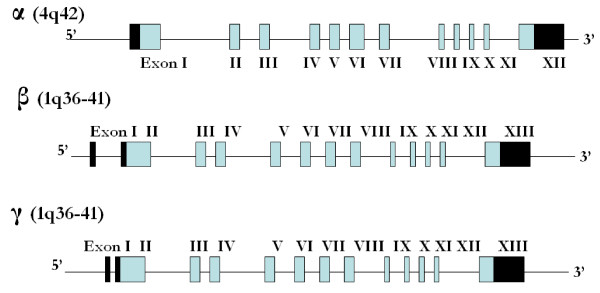
**Genomic Organization of rat ENaC α, β, and γ subunits**. At the genomic level, ENaC α, β, and γ subunits are encoded by three different genes located on separate chromosomes. The gene encoding the α ENaC subunit [Scnn1a] is located on chromosome 4q42, while the β and γ genes [Scnn1b and g] are located at a close proximity from each other on chromosome 1q36-q41 [RGD: Rat Genome Database]. α ENaC is composed of 12 exons, whereas each of the β and γ ENaC genes are composed of 13 exons. Translation starts in exon 1 for α ENaC and starts in exon 2 for β and γ ENaC. Translation ends in exon 12 for α ENaC and in exon 13 for β and γ ENaC. Therefore the 5'untranslated region [UTR] is included in exon 1 of α ENaC and in exons 1 and 2 of β and γ ENaC genes, while the 3'UTR is included in exon 12 of α ENaC and exon 13 in each of β and γ ENaC. Light shaded boxes represent the translated regions, while the black boxes represent the 3' and 5' UTR.

At the genomic level, ENaC α, β, and γ protein subunits are encoded by three different genes located on separate chromosomes. The gene encoding the α ENaC subunit [Scnn1a] is located on chromosome 4q42, while the β and γ genes [Scnn1b and g] are located at a close proximity from each other on chromosome 1q36-q41. The genomic organization of ENaC genes is found in figure 2.

## Significance of α ENaC versus β and γ ENaC

Of the three ENaC subunits, the α ENaC alone is critical to the formation of a functional channel. This is because the expression of α ENaC alone in Xenopus oocytes confers a low amiloride-sensitive sodium current, whereas neither the β nor the γ subunits can form conducting functional channels when expressed alone in Xenopus oocytes. β and γ ENaC only serve to maximize channel activity [15,22].

The critical role of α ENaC is highlighted not only in expression studies in Xenopus oocytes, but also by knockout mice models. α ENaC knockout mice died within 40 hours of birth because of water-clogged lungs and failure of fluid clearance [23]. Moreover, decreased α ENaC expression [without necessarily knocking out α ENaC] predisposes animals to a respiratory distress syndrome [24]. The β and γ subunits have only minor effects on lung fluid clearance. Owing to the critical role of α ENaC in the functionality of the channel, and the fact that it is the only ENaC subunit with currently published alternatively spliced forms in Dahl rats, α ENaC regulation by alternative splicing will be discussed in the current review.

## Critical role of ENaC in kidneys of Dahl rats

Dahl rats serve as good candidates for studying ENaC. Dahl rats are separated into two strains; the salt-sensitive [S] and the salt-resistant [R] strain because of the inherent genetic propensities of Dahl S, but not R rats to develop hypertension on high salt intake [25,26]. Renal cross-transplant studies demonstrated the decisive role of the kidneys in regulating blood pressure in Dahl S rats on regular salt diet. Indeed, Morgan et al. were able to demonstrate clearly that Dahl R rats when receiving an R kidney did not develop hypertension on high salt diet, but did with an S kidney [27]. This highlighted the critical role of the kidney in salt-sensitive hypertension in Dahl S rats.

Additionally, in vitro studies do indicate that Dahl S rats exhibit enhanced Na^+ ^transport related to ENaC. This is because monolayers of inner medullary collecting duct cells when cultured in vitro and then examined electrophysiologically showed twice the rate of Na^+^-transport when obtained from S *versus *R rats. This increase in sodium transport related to ENaC in Dahl S *versus *R rats is apparently due to a primary increase in the conductive permeability of the apical membrane to Na^+^. The authors concluded that ENaC is intrinsically different or differently regulated in kidneys of S and R rats [12].

To date, there are few reports on the regulation of ENaC in these rat models. Aoi et al. just recently reported an abnormal increase in α ENaC mRNA [2.5-fold] in the kidneys of Dahl S rats on high *versus *regular salt intake for 4 weeks [28], while Dahl R rats showed a decrease in α ENaC mRNA [29]. Changes in ENaC protein abundance have not been reported, which is important since ENaC undergoes extensive post-translational regulation.

## ENaC Differential Regulation in Dahl S versus R rats

It is essential to recognize that ENaC mutations might be the reason behind the enhanced α ENaC expression and overall ENaC activity in Dahl S *versus *R rats. A comprehensive ENaC α, β, and γ screening study is worthwhile to rule out or rule in the contribution of genetic mutations in the enhanced overall ENaC activity in Dahl S *versus *R rats. On the other hand, lack of mutations in ENaC genes in Dahl S and R rats will leave us with poorly understood mechanisms behind the enhanced ENaC activity in Dahl S *versus *R rats. An additional potential strategy for the differential ENaC regulation in Dahl rats - besides sequence variability of ENaC genes in Dahl S *versus *R rats- is via alternative splicing for the principle α ENaC subunit, which is the focus of the present review. Although it is accepted that ENaC activity is dynamically modulated by regulation of channel trafficking to the luminal membrane, little is actually known about the cellular control points and queues impinging upon this modulation. In addition, interactions of ENaC with ENaC alternatively spliced forms and the outcome of such interactions on channel-subunits expression is important for channel assembly, localization to the luminal membrane and activity, and yet remains nebulous.

## Regulation of α ENaC by alternative splicing: What is currently known?

**Figure 3:**
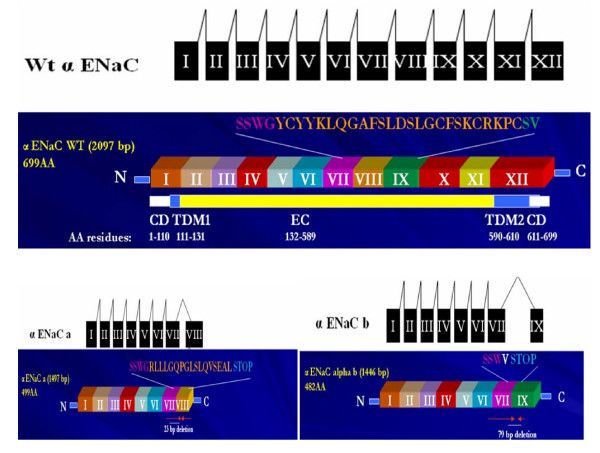
**α ENaC alternatively spliced forms**. **A schematic illustration of alternative mRNA splicing of α ENaC wildtype, -a and -b forms**. α ENaC wildtype is made of 12 exons, while α ENaC-a is formed of exons I to VIII, with a 23 bases deleted from exon VIII. On the other hand, α ENaC-b is formed of exons I to IX with a skipping of exon VIII [79 bases]. Underneath each mRNA splicing comes the protein organization of the 2 alternatively spliced forms of α ENaC [α ENaC-a & -b] that have been published in rats [33] in comparison to α ENaC wildtype major transcript. α ENaC wildtype is 698 amino acids in length [2100 bp]. Amino acid residues from 1 to 110 reside in the cytoplasm, amino acid residues from 111 to 131 constitutes the first transmembrane domain, residues 132 to 589 constitute the extracellular loop, residues 590 to 610 constitute the second transmembrane domain, and residues 611-698 are cytoplasmic. α ENaC-a alternatively spliced form is formed by the deletion of 23 nucleotides from exon 8, whereas α ENaC-b is formed by the deletion of 79 nucleotides that involved exon 8 skipping. These deletions introduced a premature stop codon and resulted in shorter proteins at the carboxy terminus by 199 in α ENaC-a and 216 amino acids in α ENaC-b, making α ENaC-a 499 amino acids [2077 bp] and α ENaC-b 482 amino acids [2021 bp] in length. These resultant shorter proteins lacked the second transmembrane domain [TDM2] which is important in channel pore formation. α ENaC-a alternatively spliced form is a low abundance transcript that is expressed in the rat kidney, tongue epithelia and tongue taste tissues. α ENaC-a binding with the channel blocker [phenamil] was greatly enhanced. This demonstrates that the amiloride-binding site [i.e ENaC blocker site] resides in the extracellular loop of the channel and not the second transmembrane domain that is presently missing in α ENaC-a [CD: cytoplasmic domain, TDM1: transmembrane domain M1, EC: extracellular loop, TDM2: transmembrane domain M2].

**Figure 4:**
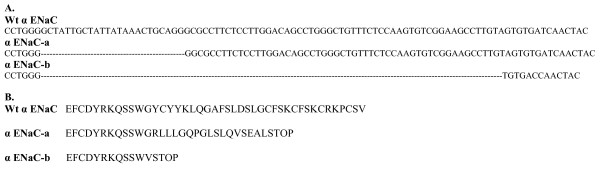
**Schematic representation of wildtype and the alternatively spliced forms α ENaC-a & -b**. **A. The genomic sequence of α ENaC wildtype, -a and -b forms**. The alternatively spliced forms α ENaC-a & -b share the same splicing site [CCTGGG] which is located within exon VII. α ENaC-a & -b had 23 and 79 bases deleted respectively resulting in the formation of a premature stop codon. **B. The protein sequence of α ENaC wildtype, -a and -b forms**. The deletions of 23 and 79 bases respectively in α ENaC-a & -b introduced a premature stop codon and resulted in shorter proteins at the carboxy terminus by 199 in α ENaC-a and 216 amino acids in α ENaC-b, making α ENaC-a 499 amino acids [1497 bp] and α ENaC-b 482 amino acids [1446 bp] in length. The α ENaC-a and -b truncated proteins of approximately 55 and 53 kDa respectively, are identical to wildtype α ENaC up to amino acids 481 and 480 respectively, followed by 17 and 1 novel amino acids unique to the spliced form after which the stop codon terminates translation (adapted with permission from reference  [33]).

Naturally occurring alternatively spliced forms have been reported for the α ENaC [not the β, or γ ENaC] in humans [30], mice [31], and chicken [32] suggesting that alternative RNA splicing is most likely a mechanism regulating α ENaC activity. To date, there are two alternatively spliced forms [α ENaC-a and -b] of the α ENaC subunit that are currently published in rats [33,34]. α ENaC-a and -b are identified in the rat kidney and tongue taste tissues [33]; [34]. The exon-intron organization of these two alternatively spliced forms are found in figures 3 and 4. The potential biological role of these alternatively spliced forms in ENaC regulation prior to and after salt loading in Dahl S rats is yet to be examined. Interestingly the 5' donor splice site [CCTGGG] used to create the α ENaC-a and -b was also utilized to create the α ENaC +22 splice variant in humans [30] and the 3399 bp variant in chicken [32]. This conservation for the 5' splice site across species underscores the significance of α ENaC-a and -b spliced forms in ENaC regulation.

Prior to an in depth discussion of the α ENaC alternatively spliced forms, we need to discuss the α ENaC subunit in depth. α ENaC is composed of 698 amino acid with a molecular weight of 78.8 kDa. Amino acid residues from 1 to 110 reside in the cytoplasm, amino acid residues from 111 to 131 constitutes the first transmembrane domain, 132-589 constitute the extracellular loop, 590-610 constitute the second transmembrane domain, and 611-698 are cytoplasmic. The exon-intron structure of α ENaC is found in figure 2.

α ENaC-a transcript is a low abundance transcript compared to full length α ENaC and has been studied in terms of expression, functionality and binding to ENaC blocker [33]. On the other hand, α ENaC-b is yet to be characterized. α ENaC-a alternatively spliced form is formed by the deletion of 23 nucleotides from exons 7 and 8 [33,34]. This deletion introduced a premature stop codon and resulted in a shorter protein at the carboxy terminus by 199 in α ENaC-a. This resultant shorter protein lacked the second transmembrane domain which is important in channel pore formation. The α ENaC-a form alone has been studied in depth in terms of expression, tissue distribution, functionality in vivo and binding with the phenamil compound [phenamil is a derivative of amiloride and acts as a channel blocker] [33,34]. α ENaC-a alternatively spliced form is expressed in the rat kidney, tongue epithelia and tongue taste tissues enriched in circumvallate papillae. Regarding functionality, α ENaC-a failed to generate amiloride sensitive Na^+ ^current when expressed in Xenopus oocytes, but still retained binding with the channel blocker [phenamil] that was greatly enhanced. This demonstrates that the amiloride-binding site resides in the extracellular loop of the channel and not the second transmembrane domain.

On the other hand, α ENaC-b formation involves exon 8 skipping [79 nucleotides]. α ENaC-b is a truncated protein of 53 kDa that is identical to full length α ENaC up to amino acid 480, followed by one novel amino acid unique to α ENaC-b after which the stop codon terminates translation. α ENaC-b lacks the second transmembrane domain which is critical in channel pore formation [figure 1]. The significance of the second transmembrane domain is highlighted by the presence of the ENaC selectivity filter [that allows for a high selectivity for Na^+^] in a region localized to a three-residue [G/SxS] track immediately preceding the second transmembrane domain of the ENaC subunits. This track resides in the narrowest part of the pore to exclude all, but the smallest cations. The three-residue track is located at amino acid 587, 529, 534 for ENaC α, β, and γ respectively [35]. Not only does TM2 control ion selectivity, but also contribute to ion permeation. Point mutations of selected residues within TM2 particularly amino acids 595 and 602 reduced Na^+ ^currents significantly and allowed for K^+ ^permeation over Na^+ ^permeation [36]. Owing to the critical role of the second transmembrane domain, α ENaC-b is expected to be a non functional transcript similar to α ENaC-a that previously failed to generate amiloride sensitive Na^+ ^current when expressed in Xenopus oocytes [33]. Because of the non functionality of all α ENaC alternatively spliced forms in all species, it has been proposed to act as dominant negatives on the wildtype α ENaC. The genotoxic effects of alternatively spliced forms have been widely reported in several proteins, channels and membrane receptors and will be highlighted in section VI.

In agreement with the potential significance of alternative splicing in regulating ENaC, Xu et al., demonstrated a suppression in α ENaC spliced forms by oxidative stress in the lung epithelial cells in humans [37]. This finding is critical for ENaC regulation in Dahl rats because of the remarkable oxidative stress levels reported in most of the tissues of high-salt-fed Dahl S rats [38-40].

Our current review examines two potential mechanisms by which α ENaC spliced forms regulate the renal full length α ENaC possibly by a dominant negative effect. The **first mechanism **is through the enhanced binding of α ENaC spliced forms protein to the full length α ENaC. Enhanced binding of α ENaC spliced forms to the full length α ENaC might in itself hinder proper channel assembly and interfere with proper channel activity. It might as well facilitate ENaC degradation in the cytoplasm and/or inhibit proper ENaC insertion into the plasma membrane, and therefore, contribute to the formation of non functional channels. The **second mechanism **is through an enhanced degradation of full length α ENaC as a result of direct binding to α ENaC spliced forms, a phenomenon that has been reported previously in several other channels and membrane proteins [41-46].

## Implications of alternative splicing of ion-channels: Possible confounders in the "Genotoxicity" of α ENaC alternatively spliced forms

Alternative splicing is a regulated process that takes place when the exons of a certain pre-mRNA are spliced in more than one way to yield several possible mature mRNAs from a single gene. 59% of human genes have more than one splice form [47], and 80% of alternative splicing changes the encoded protein [48]. The functional significance of ion channel-alternatively spliced forms vary considerably from altering the channel activation and inactivation rates for K^+ ^channels [49,50], to altering gating properties for Ca^++ ^channels [51], unit conductance, ion selectivity or sensitivity [52], or fine physiological tuning for optimal tissue performance [41,53].

Aside from the fact that alternative splicing is a major contributor to the structural and functional diversity of ion channels such as the Na^+^, K^+^, and Ca^++ ^channels, a major surge of interest has been recently witnessed in the genotoxic potential of alternatively spliced forms on full length forms [41-45]. Often, dominant negative alternatively spliced forms sequester the full length form in the cytoplasm and subsequently enhance its proteolytic degradation, such an intriguing phenomenon that greatly emphasize the importance of alternative splicing in physiology, development and disease [54-57].

**Figure 5:**
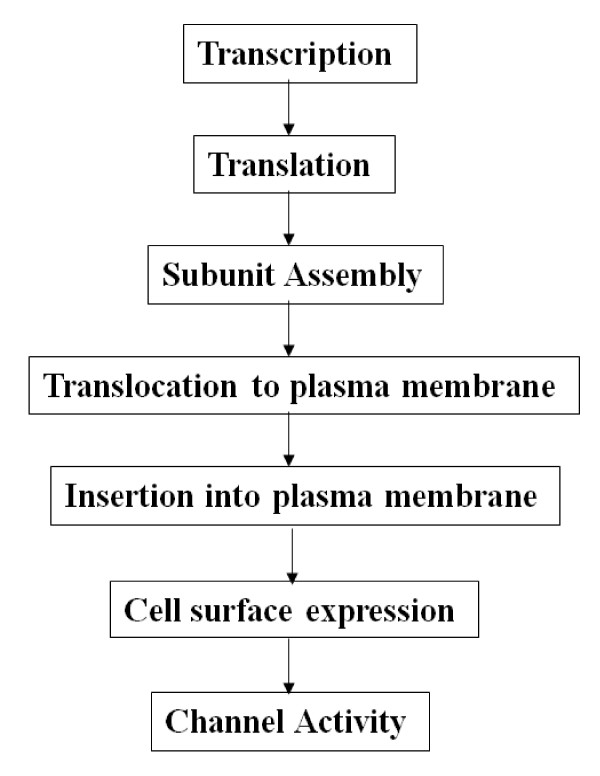
**Control points impinging on ENaC cell surface expression and activity**. Schematic representation of the steps involved in regulating α ENaC cell surface expression and activity. These steps include α ENaC subunit synthesis, assembly with the β and γ subunits, trafficking, insertion into the plasma membrane and activity. α ENaC-b may hinder any of these steps causing a suppressed channel cell surface expression and/or activity in Dahl R *versus *S rats that is augmented on high salt diet.

Moreover, the biological impact of alternatively spliced forms, particularly those lacking functional domains such as the second transmembrane domain in α ENaC-a and -b, may go as far as a switch-off effect [58]. However, one might wonder if the "genotoxicity" of a given spliced form is exerted mainly at the expense of full-length transcription and/or translation [potentially by accelerating full-length proteolytic degradation]; or if it is primarily impacting channel assembly and/or translocation to the plasma membrane; or if it solely hinders channel cell surface expression and/or activity? [Figure 5].

Alternatively spliced forms have been shown to impair any of the above mentioned cellular processes, either independently or in synergy. For example, a spliced form of the K^+ ^channel [SV1] was shown to impair full-length translation, subunit assembly, translocation to the plasma membrane, cell surface expression and activity. SV1 specifically inhibited cell surface expression of the full-length K^+ ^channel α or β subunits by ~80%, by trapping them in the endoplasmic reticulum [ER] [41]. SV1, in turn, prevented subunit trafficking to the plasma membrane because of retention in the endoplasmic reticulum. Moreover, SV1 diminished protein expression of the K^+ ^channel subunits and cells that express it failed to generate a current.

Similarly, splice variants of the cation-channel [TRPV4] impaired subunit oligomerization, enhanced subunit accumulation in the endoplasmic reticulum and hindered trafficking [59]. Additionally, a spliced form of the inhibitor of apoptosis protein family was shown to be expressed at mRNA levels that were 2-3% of the levels of the full-length transcript, yet it encodes a protein that accumulates 50-fold higher levels than full-length and this accumulated protein competes with full-length form for activity [60]. Likewise, co-expression of the calcium sensing receptor splice variant with its full-length form reduced the expression and activity of the full-length form in a dose-dependent fashion [61]. Consistent with the notion of a so-called "dominant negative" effect of alternatively spliced forms on full-length forms, a spliced form of the GTP cyclohydrolase enzyme [GCH] suppressed full-length GCH form expression levels in a dose-dependent manner, possibly by heteromeric interactions that ultimately decreased the stability and activity of the full-length form [45].

As such, common findings that support the role of a spliced form as a dominant negative expression regulator can be summarized as follows: a] the spliced form is non-functional [42], b] the frequency of the spliced form is higher relative to the full-length form; c] the spliced form heterodimerizes with the full length form [45]; and finally d] the spliced form accelerates full-length form degradation by trapping the latter in the endoplasmic reticulum [42].

## Conclusion and future perspectives

Owing to the fact that alternative splicing is a strictly regulated process, and that alternatively spliced forms either serve as important regulators for the parent gene, possibly by a dominant negative effect, or as diagnostic markers for several pathological states particularly human genetic diseases, therefore, this review was meant to highlight recent findings with regards to the putative mechanism by which α ENaC alternatively spliced form [s] modulate ENaC activity in response to high salt diet in Dahl-S *versus *R rats. Understanding the significance of α ENaC alternative splicing in modulating ENaC in kidneys of Dahl rats is worthwhile because of the enhanced ENaC activity in Dahl S *versus *R rats.

Knowledge of the mechanism by which α ENaC spliced forms regulate full length α ENaC and possibly prevent the hyperactivity of ENaC in Dahl S rats and the subsequent genesis of salt-dependent hypertension [a disease that comprises a large subgroup [over 50%] of Canadian adults] would certainly enhance the understanding of the basic regulation of ENaC and the pathophysiology of ENaC-associated disorders such as salt-sensitive hypertension. It may also create one or more specific targets for the development of novel anti-hypertensive drug or gene therapy.

## Competing interests

The author declares that they have no competing interests.

## Authors' contributions

MS wrote, finalized and approved the current manuscript.
